# Duodenal mucosal RNA-Seq identifies coordinated bile acid–axis transcriptional alterations in food-responsive enteropathy in dogs

**DOI:** 10.3389/fvets.2026.1829399

**Published:** 2026-06-11

**Authors:** Borbála Mózes, Gergely Kiss, Ábel Fóthi, Szilamér Ferenczi, Roland Psáder

**Affiliations:** 1 Department of Internal Medicine, University of Veterinary Medicine Budapest, Budapest, Hungary; 2 Department of Molecular Biology, Institute of Biochemistry and Molecular Biology, Semmelweis University, Budapest, Hungary; 3 Laboratory of Molecular Neuroendocrinology, Institute of Experimental Medicine, Hungarian Academy of Sciences, Budapest, Hungary

**Keywords:** bile acid signaling, canine chronic inflammatory enteropathy, CIE, FGF19, food-responsive enteropathy, FXR, RNA-Seq, TGR5

## Abstract

Food-responsive enteropathy (FRE) is a common form of chronic inflammatory enteropathy in dogs. Its underlying molecular mechanisms remain incompletely characterized. Increasing evidence from human studies and emerging canine data suggests that bile acids (BAs) influence intestinal homeostasis and inflammation. Duodenal mucosal biopsies from dogs with FRE (*n* = 8) and healthy controls (*n* = 4) were analyzed by bulk RNA sequencing. Differential expression analysis (DESeq2), KEGG and Reactome pathway enrichment, and GSEA were performed with a specific focus on BA transport, sensing, and metabolic pathways. FRE samples showed a distinct BA-associated transcriptional signature, including a non-significant decreasing trend of the BA receptor NR1H4 (FXR) expression (padj = 0.057), and significant upregulation of the nuclear receptor RXRA, together with increased expression of downstream mediators NR0B2 (SHP) and FGF19. BA transport components such as SLC51A (OSTα) and ABCC3 (MRP3) were differentially regulated, and the BA-synthetic enzyme HSD3B7 was increased. Bile secretion was among the top enriched KEGG pathways (ranked 9th; NES = 1.935; padj = 0.005). This study provides, to our knowledge, the first focused mucosal transcriptomic evidence of coordinated BA-axis alterations in canine FRE. The findings align with mechanisms described in human inflammatory bowel disease and support further investigation of bile acid signaling in canine chronic enteropathy. Findings should be interpreted as exploratory due to cohort heterogeneity.

## 1 Introduction

Canine chronic inflammatory enteropathy (CIE) is a common cause of persistent gastrointestinal signs in dogs, including chronic diarrhea, vomiting and weight loss. Classification is traditionally based on treatment response, distinguishing food-responsive enteropathy (FRE), immunosuppressant-responsive enteropathy (IRE) and non-responsive enteropathy (NRE). These disease phenotypes can also be protein-losing enteropathies (PLE) (1–5). Among non–protein-losing forms, FRE represents the largest subgroup and is characterized by complete clinical remission following a diet change to a therapeutic diet (3, 4). Empirical antimicrobial therapy is no longer recommended for chronic enteropathies unless specific indications are present (4, 5).

Bile acids (BAs) are increasingly recognized as key modulators of intestinal homeostasis. Besides their classic role in lipid absorption, BAs act as signaling molecules influencing epithelial integrity, immune activation, and gut microbial composition (6–9). In human inflammatory bowel disease (IBD), altered BA composition and impaired FXR-mediated signaling are associated with mucosal inflammation and barrier dysfunction (8–13).

Similar patterns have been observed in canine chronic inflammatory enteropathies. Dogs with CIE display altered fecal BA profiles, reduced secondary BA concentrations, and microbial shifts affecting BA metabolism (14, 15). Secondary BAs have demonstrated anti-inflammatory effects in canine macrophages (16), and intestinal organoid studies from dogs with IBD reveal transcriptional disturbances in BA-related and lipid metabolic pathways (17, 18). Reduced ileal expression of the apical sodium-dependent bile acid transporter (ASBT) has been reported in dogs with CIE, suggesting impaired ileal BA reabsorption (19). These findings collectively support a role for BA dysregulation in canine intestinal disease, yet the specific mucosal expression patterns of BA transporters, receptors, and regulatory elements in FRE remain unknown.

Key components of BA metabolism—including apical uptake transporters (SLC10A2/ASBT), which has been reported to be reduced in dogs with chronic enteropathies (19), intracellular BA-binding proteins (FABP6/ILBP), basolateral efflux transporters (SLC51A/B, ABCC3, ABCC4), and signaling mediators such as FXR (NR1H4), RXRA, NR0B2 (SHP), FGF19, and GPBAR1 (TGR5) – have gained significant scientific attention in the realm of human intestinal and systemic health and disease (20–26), but have not been systematically characterized at the transcriptomic level in dogs with FRE. Studies in humans and rodent models indicate that disruptions in these pathways are associated with intestinal inflammation and altered epithelial function (22, 24, 27–31). Whether comparable mechanisms operate in canine FRE has not yet been investigated.

To address these gaps, we applied an exploratory bulk RNA sequencing approach to duodenal mucosal biopsies from dogs with food-responsive enteropathy (FRE) and healthy controls. Pathway-level analyses identified multiple altered biological processes, including metabolic, mitochondrial, epithelial barrier, and immune-related pathways, consistent with previous transcriptomic studies in canine chronic enteropathies (32). Notably, several of the most strongly enriched pathways in FRE were related to cellular metabolism and energy homeostasis, whereas multiple immune-associated pathways showed relative downregulation compared with controls. Among the affected pathways, bile acid–related regulatory mechanisms emerged as a biologically coherent yet comparatively underexplored functional axis at the transcriptomic level in canine intestinal mucosal biopsies. This study was designed as a hypothesis-driven analysis focusing on bile acid–related pathways rather than a transcriptome-wide discovery approach.

Accordingly, the present short communication focuses on bile acid–associated transcriptional changes as an interpretative subset of the broader exploratory dataset.

## 2 Materials and methods

### 2.1 Animals and study design

Dogs with chronic gastrointestinal signs (>3 weeks duration) were prospectively enrolled at the University of Veterinary Medicine Budapest. Inclusion criteria consisted of:

(1) Persistent gastrointestinal signs such as vomiting, diarrhea, or weight loss; (2) Exclusion of extraintestinal, infectious, metabolic, and neoplastic causes through routine diagnostic evaluation; (3) No antibiotic or immunosuppressive treatment within 30 days prior to endoscopy (3, 4). FRE was diagnosed based on clinical remission following a hypoallergenic diet trial (feeding a novel protein source or hydrolyzed diet exclusively) that was maintained for at least a month. Eight client-owned mixed and purebred dogs met the diagnostic criteria for FRE, none of the enrolled FRE dogs were hypoalbuminemic at the time of biopsy collection. Healthy controls consisted of four purpose-bred research beagles (ATRC Aurigon Toxicology Research Center) without history or clinical evidence of gastrointestinal disease. Detailed clinical summaries are provided in Supplementary Table S1.

### 2.2 Endoscopic sampling and tissue handling

Upper gastrointestinal endoscopy was performed under general anesthesia following standard clinical protocols. Mucosal biopsies were collected from the stomach and proximal duodenum in all dogs, and from the ileum and large intestine when both upper and lower endoscopy were indicated. For transcriptomic analysis, only duodenal biopsies were used. Freshly collected biopsies were immediately immersed in RNAprotect Tissue Reagent (Qiagen), incubated at 4 °C overnight according to the manufacturer’s instructions, and subsequently stored at −80 °C until RNA extraction. Parallel biopsies were submitted for routine histopathology following WSAVA guidelines (1, 33). Mild to severe lymphoplasmacytic and/or eosinophilic infiltration was detected in all FRE duodenal samples, whereas control samples showed inflammatory cell abundance within normal limits.

### 2.3 RNA extraction and quality control

Total RNA was extracted using the RNeasy Mini Kit (Qiagen), including on-column DNase I digestion to remove genomic DNA contamination. RNA concentration and integrity were assessed using Qubit fluorometry and LabChip GX Touch capillary electrophoresis, and only samples meeting predefined quality criteria were used for library preparation.

### 2.4 Library preparation and sequencing

Polyadenylated RNA was enriched, and strand-specific sequencing libraries were generated using the NextFlex Rapid Directional RNA-Seq kit 2.0 (PerkinElmer). Libraries were sequenced on an Illumina NovaSeq 6,000 platform (2 × 150 bp paired-end reads), targeting >50 M paired-end reads per sample. Quality filtering removed bases with Phred score < 30, reads shorter than 50 bp, and reads with mean base quality < 30.

### 2.5 Bioinformatic workflow

FASTQ files were processed using the RaNA-seq online platform (34). Alignment to CanFam3.1 reference genome and gene-level quantification were performed using Salmon via RaNA-seq (35). Differential gene expression was assessed by comparing the two groups (FRE vs. healthy controls) using DESeq2. Given collinearity between group and variables such as age and breed, and the limited sample size, inclusion of covariates was not statistically appropriate. Sequencing quality metrics, including library size and mapping rates, were consistent across samples, with no outliers or systematic differences observed between groups (Supplementary Table S2).

### 2.6 Pathway enrichment analysis

Functional interpretation included over-representation analysis and gene set enrichment analysis (GSEA) using KEGG, Reactome, and WikiPathways gene sets implemented in RaNA-seq. GSEA was performed using DESeq2 rank statistics implemented in RaNA-seq, and significance was assessed based on nominal *p*-values (34). Enrichment analysis was performed using the full list of genes ranked by statistical significance, without applying an additional fold change cutoff. In addition, an *a priori* bile acid–related gene set comprising twelve genes was defined based on KEGG annotations and published CIE/IBD studies (15–18, 22, 25, 26, 28, 30–32, 36).

### 2.7 Statistical analysis

Differentially expressed genes (DEGs) were considered statistically significant at an adjusted *p*-value (padj) < 0.05 (Benjamini–Hochberg correction). No strict log2 fold change threshold was applied for defining differentially expressed genes. Given the exploratory nature of the study and the focus on regulatory pathways, genes were selected based on adjusted *p*-values, while log2 fold changes are reported for transparency. Due to the clinical nature of the cohort, variables such as age, breed, and environmental exposure were not balanced between groups and may be confounded with disease status. PCA was performed on the full transcriptome and interpreted descriptively. PCA coordinates (PC1 and PC2) and corresponding sample-level metadata are provided in Supplementary Table S3. Correlation between principal components and potential confounders were assessed using Spearman’s rank correlation due to the small sample size and potential non-normality of PCA coordinates. All samples met the predefined RNA quality threshold required for sequencing, and all samples were processed using the same experimental workflow, minimizing potential batch effects.

## 3 Results

### 3.1 Global transcriptional patterns

Sequencing quality metrics were consistent across samples, with library sizes (total fragments) and mapping rates (68.8–71.1%) showing no outliers. GC content showed minimal variation across samples (~49–51%) and did not correlate with principal component scores (Spearman correlation: PC1 *ρ* = 0.43, *p* = 0.28; PC2 ρ = 0.00, *p* = 1.0), indicating that sequence composition bias did not contribute to sample clustering (Supplementary Table S2). PCA based on the full transcriptome revealed separation between duodenal mucosal samples from dogs with FRE and healthy controls along PC1 (65.76% variance explained). FRE samples formed a relatively compact cluster despite heterogeneity in age, breed, and clinical background, whereas variability was also present among control samples (Supplementary Figures S1A–C). Within the FRE cohort no gradient-like distribution of samples along principal components was observed with respect to age or body weight. Consistently, within the FRE group, no significant correlations were detected between age and PC1 (*ρ* = 0.19, *p* = 0.65) or PC2 (ρ = −0.52, *p* = 0.19), nor between body weight and PC1 (ρ = −0.14, *p* = 0.74) or PC2 (ρ = 0.29, *p* = 0.49). The observed associations are mostly weak and non-significant. These findings suggest that age and body weight are unlikely to be dominant drivers of the observed sample distribution. Given the limited sample size, variability should be interpreted cautiously.

Global differential expression patterns are shown in a volcano plot (Supplementary Figure S2).

Exploratory pathway enrichment identified multiple altered biological processes (Supplementary Table S4). Among the top positively enriched KEGG pathways in FRE were metabolic and cellular homeostasis–related pathways, including citrate cycle (TCA cycle), carbon metabolism, AMPK signaling, mitophagy, peroxisome, and tight junction pathways. In contrast, several immune-associated pathways, including inflammatory bowel disease (IBD), cytokine–cytokine receptor interaction, intestinal immune network for IgA production, NF-kappa B signaling, and Th1/Th2 and Th17 differentiation pathways, showed relative downregulation compared with controls. Detailed interpretation of these global pathway patterns was beyond the focused scope of the present short communication, which primarily concentrated on bile acid–related pathways. Notably, bile secretion was among the top enriched pathways (ranked 9th; NES = 1.935, padj = 0.005). To assess potential confounding by age and body weight, subsampling strategies aimed at improving group comparability were performed (Supplementary Table S5). Across subsets, PCA-based group separation was retained, although increased variability was observed in smaller subsets. Importantly, bile acid–related pathway signals remained detectable, either by ORA in the less restrictive subset or by GSEA in the most restrictive subset, consistent with reduced statistical power rather than absence of the underlying signal.

### 3.2 Enrichment of bile acid-related pathways

Pathway-level analyses identified BA-related pathways among the altered functional modules in dogs with FRE.

Specifically, the KEGG Bile secretion pathway (cfa04976) was significantly enriched (NES = 1.935, padj = 0.005). In addition, Reactome BA-related pathways, including Synthesis of bile acids and bile salts (R-CFA-192105), were also overrepresented together with additional bile acid–associated functional categories identified in overrepresentation analysis.

Within the KEGG Bile secretion pathway (cfa04976), six core bile acid–related genes detected in the dataset (NR1H4, RXRA, NR0B2, ABCC3, ABCC4, and SLC51A) showed consistent directional changes in FRE (Supplementary Figure S3). Among the remaining genes, HSD3B7 corresponded to the enzymatic activity annotated in the KEGG primary bile acid biosynthesis pathway (cfa00120; EC 1.1.1.181) (Supplementary Figure S4).

FGF19 was not included in either KEGG bile acid pathway, reflecting its role as an enterohepatic feedback mediator rather than a structural component of bile acid synthesis or transport.

These findings suggest that BA metabolism and sensing pathways represent a particular component of the broader transcriptional alterations observed in FRE.

### 3.3 Coordinated transcriptional alterations in bile acid regulatory genes

Based on functional annotation, core genes involved in bile acid sensing, transport, and downstream regulatory signaling in intestinal epithelial cells were selected for focused transcript-level analysis.

Transcript-level analyses revealed coordinated changes of multiple components involved in bile acid transport, sensing, and downstream signaling (Table 1). Several transcripts reached statistical significance after multiple testing correction, while others showed consistent directional trends. Specifically, the bile acid receptor FXR (NR1H4) showed a non-significant reduction in expression in FRE compared with controls (log2FC = −0.34; padj = 0.057), while its obligate heterodimerization partner RXRA was significantly upregulated (log2FC = 0.41; padj = 0.014). Key FXR-induced genes were also increased, including NR0B2 (SHP) (log2FC = 1.10; padj = 0.00003) and FGF19 (log2FC = 1.89; padj = 0.00016). In parallel, components of the bile acid transport machinery were altered, with increased expression of SLC51A (OSTα) (log2FC = 0.81; padj = 0.011) and ABCC3 (MRP3) (log2FC = 0.77; padj = 0.0039), together with decreased expression of ABCC4 (MRP4) (log2FC = −0.59; padj = 0.0009). In addition, the bile acid biosynthetic enzyme HSD3B7 was significantly upregulated (log2FC = 0.85; padj = 0.0054).

**Table 1:** Differential expression of selected bile acid-associated genes in the duodenal mucosa of dogs with food-responsive enteropathy (FRE) compared with healthy controls.

Category	Gene	Symbol/common name	Description	log2FC	padj	Direction
Bile acid transport	ABCC3	MRP3	FXR-induced, expressed in intestine	0.77	0.00391	↑ up
Bile acid transport	ABCC4	MRP4	FXR-induced, expressed in intestine	−0.59	0.0009	↓ down
Bile acid transport	SLC10A2	Solute carrier family 10 member 2	Active in ileum and duodenum	0.09	0.888	↑ up
Bile acid transport	SLC51A	OSTα	FXR-induced, expressed in intestine	0.81	0.0114	↑ up
Bile acid transport	SLC51B	OSTβ	FXR-induced, expressed in intestine	0.09	0.806	↑ up
Bile acid sensing/signalling	NR1H4	FXR	Key receptor, active in intestine	−0.34	0.0571	↓ down
Bile acid sensing/signalling	GPBAR1	TGR5	Active in enteroendocrine cells	−0.53	0.122	↓ down
Bile acid sensing/signalling	RXRA	Retinoid X receptor alpha	FXR partner, expressed in intestine	0.41	0.0142	↑ up
Bile acid sensing/signalling	NR0B2	SHP	FXR target gene, active in intestine	1.10	0.0000323	↑ up
Regulation of bile acid synthesis	FGF19	Fibroblast growth factor 19	FXR-induced hormone, expressed in duodenum	1.89	0.000164	↑ up
Bile acid conjugation/modification	HSD3B7	Hydroxysteroid 3-beta dehydrogenase type 7	Low-level expression in intestine	0.85	0.00536	↑ up
Bile acid reuptake	FABP6	Fatty acid-binding protein 6	Predominant in ileum, present in duodenum	0.35	0.298	↑ up

This table shown are gene categories, gene symbols, functional annotations, log2 fold changes (log2FC), adjusted *p*-values (padj; Benjamini–Hochberg correction), and direction of change (↑ upregulated; ↓ downregulated in FRE vs. control).

**Figure 1:**
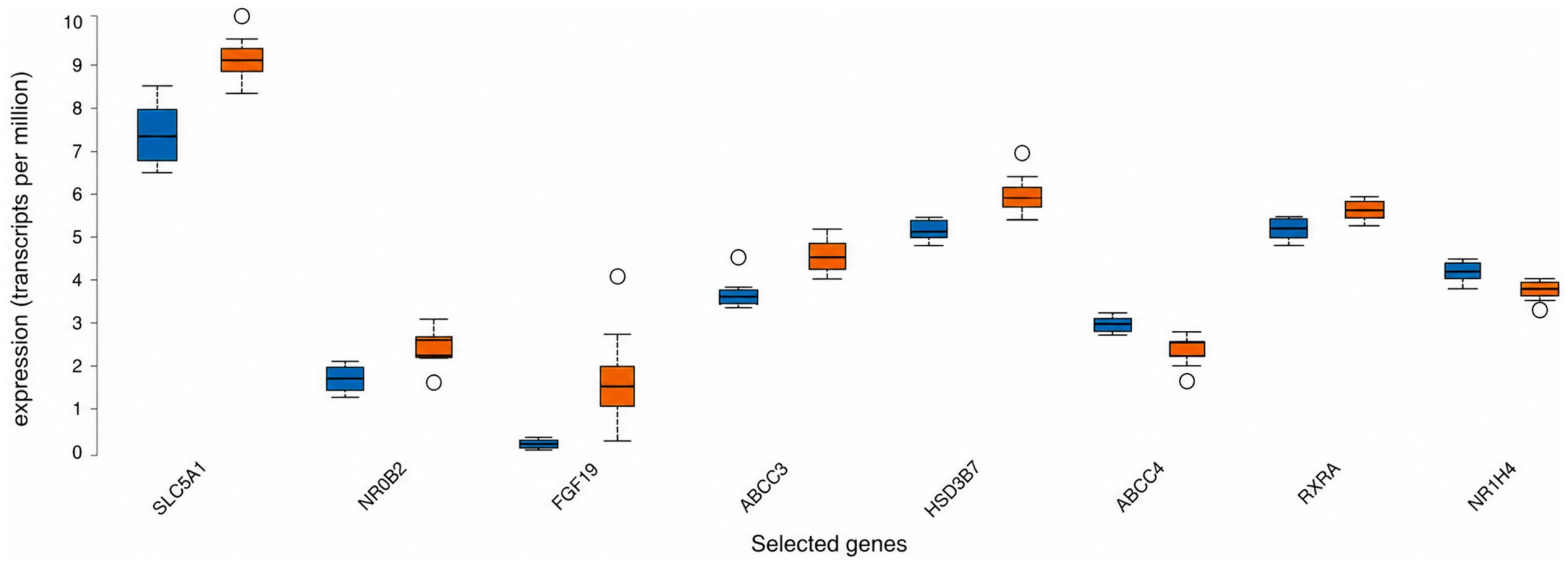
TPM-normalized expression levels of selected bile acid-associated genes in duodenal mucosal samples from dogs with food-responsive enteropathy (FRE, orange) and healthy controls (blue). Boxplots show the median, interquartile range, and individual outliers.

**Figure 2:**
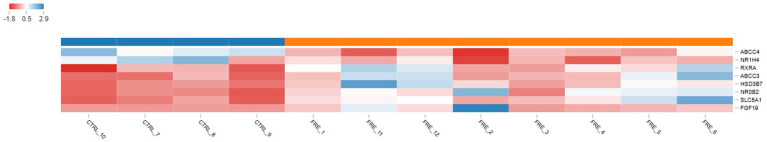
Heatmap of TPM-normalized expression values for selected bile acid-associated genes in duodenal mucosal samples from healthy controls (left, blue bar) and dogs with food-responsive enteropathy (FRE, right, orange bar). Colors represent relative expression levels (row-scaled), with red indicating lower and blue indicating higher expression. Columns correspond to individual samples.

TPM-normalized expression distributions of altered bile acid–associated genes are shown in Figure 1. These gene-wise boxplots show significantly increased expression of NR0B2, FGF19, ABCC3, HSD3B7, SLC51A, and RXRA in FRE, with decreased ABCC4, and a non-significant decreasing trend for NR1H4 (orange: FRE; blue: control). Heatmap clustering (Figure 2) indicated a structured but heterogeneous pattern across the BA-axis gene set (orange: FRE; blue: control). As this heatmap represents a targeted bile acid–related gene set, it reflects pathway-specific variability and should not be directly compared with the global transcriptomic structure captured by PCA.

Together, these alterations are consistent with coordinated transcriptional modulation within the intestinal bile acid regulatory network, with reduced NR1H4 expression and increased expression of downstream regulatory and transport components. In contrast, key bile acid–related genes involved in apical uptake and intracellular binding, including SLC10A2 (ASBT), FABP6 (ILBP), GPBAR1 (TGR5), and SLC51B, did not show statistically significant differential expression between FRE and control samples.

## 4 Discussion

The present study provides, to our knowledge, the first transcriptomic characterization of bile acid-associated pathways in the duodenal mucosa of dogs with food-responsive enteropathy. Our findings are consistent with coordinated alterations in BA transport, sensing, and regulatory signaling, suggesting involvement of BA-axis regulatory pathways in canine chronic enteropathy. Importantly, this focused interpretation was also supported by global pathway enrichment analysis, in which bile secretion was among the top enriched KEGG pathways (ranked 9th; NES = 1.935; padj = 0.005). These observations parallel several mechanisms described in human inflammatory bowel disease, including impaired farnesoid-X receptor (FXR) activation, altered BA receptor expression, and secondary changes in downstream metabolic pathways (7, 8, 11–13, 20–23). Interestingly, global pathway enrichment in FRE was characterized not only by bile acid–associated changes, but also by enrichment of metabolic, mitochondrial, and epithelial homeostasis pathways, whereas several immune-related pathways including inflammatory bowel disease (IBD) and cytokine signaling pathways showed relative downregulation compared with controls.

NR1H4 (the gene coding FXR) showed a non-significant decreasing trend in FRE (log2FC = −0.34; padj = 0.057), which should be interpreted cautiously but is directionally consistent with reports from human IBD, where reduced FXR signaling has been associated with epithelial barrier dysfunction, inflammatory cytokine production, and dysbiosis (11–13). The simultaneous upregulation of FXR-associated genes, including NR0B2 (gene coding small heterodimer partner, SHP) and fibroblast growth factor 19 (FGF19), suggests altered regulation within this signaling axis, although direct functional conclusions cannot be drawn from transcriptomic data alone. The observed increase in retinoid-X receptor alpha (RXRA), the obligate heterodimerization partner of FXR, further supports modulation of this regulatory axis.

Alterations in BA transport machinery—including increased SLC51A (OSTα) and ABCC3 (MRP3) with decreased ABCC4 (MRP4)—mirror patterns reported in human IBD and experimental models of BA-mediated mucosal injury (24–26, 29–31). These changes are consistent with epithelial regulatory adaptation to altered bile acid signaling that may modulate BA flow during inflammation (20–22, 25, 26, 31). Upregulation of HSD3B7, a key enzyme in BA biosynthesis (37), supports the concept that mucosal BA metabolism may undergo secondary remodeling in chronic enteropathy. Taken together, these changes were modest in magnitude but directionally consistent across several components of the pathway.

Notably, the change in expression of several BA-related uptake and signaling components (SLC10A2, FABP6, GPBAR1, SLC51B) did not reach statistical significance, and therefore no definitive conclusion can be drawn regarding their regulation.

Previous canine studies support a link between bile acid metabolism, dysbiosis, and mucosal immune activity (14–18). Our findings extend these observations to the duodenal mucosa of dogs with FRE and suggest that bile acid–related transcriptional changes may form part of a broader disease-associated regulatory response.

This study has several limitations. The sample size was modest, and the clinical design did not allow matching of control and FRE dogs for variables such as age, breed, diet, or environmental exposure; therefore, potential confounding effects cannot be excluded and cannot be fully resolved given the non-overlapping structure of the study groups. Subsampling analyses aimed at improving group comparability yielded qualitatively consistent PCA structure and preserved bile acid–related pathway signals across approaches. Given the limited sample size, subset analyses are inherently sensitive to sample composition, and variability in detectability is expected due to reduced statistical power rather than absence of the underlying biological signal. These sensitivity analyses suggest that the observed bile acid–related transcriptional signal is not solely driven by the inclusion of older animals.

The absence of a strict fold change threshold may include genes with modest expression changes; however, such changes can be biologically relevant in regulatory pathways and were therefore retained in this exploratory analysis.

Transcriptomic profiling was restricted to duodenal tissue, which limits assessment of bile acid transporters and regulators more prominently expressed in the ileum. In addition, several observed expression changes were moderate in magnitude, and no complementary validation at the protein, metabolite, or functional level was performed. Finally, the present work was designed as a focused short communication centered on bile acid–related pathways rather than a comprehensive transcriptome-wide mechanistic dissection. The findings should therefore be regarded as exploratory and hypothesis-generating.

Together, these results suggest coordinated transcriptional alterations in bile acid metabolism and BA-mediated signaling in canine FRE. These findings should be interpreted in the context of a pathway-focused analysis, rather than a comprehensive transcriptome-wide evaluation. Rather, bile acid–related genes represented a biologically coherent and comparatively underexplored regulatory axis within a broader transcriptional response that also included inflammatory, immune, and epithelial pathways. Future studies integrating bile acid metabolomics, receptor protein expression, and microbiome functional profiling will be required to determine the mechanistic significance of these observations.

## Data Availability

The data presented in the study are deposited in the Zenodo repository, accession number https://doi.org/10.5281/zenodo.20330471.

## References

[1] Allenspach K Mochel JP. Current diagnostics for chronic enteropathies in dogs. Veterinary Clinical Pathol. (2022) 50:18–28. doi: 10.1111/vcp.13068, 34699081

[2] Jergens AE Heilmann RM. Canine chronic enteropathy-current state-of-the-art and emerging concepts. Front Vet Sci. (2022) 9:923013. doi: 10.3389/fvets.2022.923013, 36213409 PMC9534534

[3] Dupouy-Manescau N Méric T Sénécat O Drut A Valentin S Leal RO . Updating the classification of chronic inflammatory enteropathies in dogs. Animals (Basel). (2024) 14:681. doi: 10.3390/ani14050681, 38473066 PMC10931249

[4] Cerquetella M Rossi G Suchodolski JS Schmitz SS Allenspach K Rodríguez-Franco F . Proposal for rational antibacterial use in the diagnosis and treatment of dogs with chronic diarrhoea. J Small Anim Pract. (2020) 61:211–5. doi: 10.1111/jsap.13122, 32065388 PMC7079140

[5] Heilmann RM Jergens AE Kathrani A Allenspach K Salavati Schmitz S Priestnall SL . ACVIM–endorsed statement: consensus statement and systematic review on guidelines for the diagnosis and treatment of chronic inflammatory enteropathy in dogs. J Vet Intern Med. (2026) 40:1–27. doi: 10.1093/jvimsj/aalaf017, 41742497 PMC12881957

[6] Yang J Palmiotti A Kuipers F. Emerging roles of bile acids in control of intestinal functions. Curr Opin Clin Nutr Metab Care. (2021) 24:127–33. doi: 10.1097/MCO.0000000000000709, 33075001

[7] Song G Xie Y Yi L Cheng W Jia H Shi W . Bile acids affect intestinal barrier function through FXR and TGR5. Front Med. (2025) 12:12. doi: 10.3389/fmed.2025.1607899, 40692955 PMC12277261

[8] Shi L Jin L Huang W. Bile acids, intestinal barrier dysfunction, and related diseases. Cells. (2023) 12:1888. doi: 10.3390/cells12141888, 37508557 PMC10377837

[9] Shulpekova Y Shirokova E Zharkova M Tkachenko P Tikhonov I Stepanov A . A recent ten-year perspective: bile acid metabolism and signaling. Molecules. (2022) 27:1983. doi: 10.3390/molecules27061983, 35335345 PMC8953976

[10] Ding L Yang L Wang Z Huang W. Bile acid nuclear receptor FXR and digestive system diseases. Acta Pharm Sin B. (2015) 5:135–44. doi: 10.1016/j.apsb.2015.01.004, 26579439 PMC4629217

[11] Gadaleta RM van Erpecum KJ Oldenburg B Willemsen ECL Renooij W Murzilli S . Farnesoid X receptor activation inhibits inflammation and preserves the intestinal barrier in inflammatory bowel disease. Gut. (2011) 60:463–72. doi: 10.1136/gut.2010.212159, 21242261

[12] Curley CE Lajczak-McGinley NK Adorini L Ní Chonghaile T Keely SJ. Farnesoid X receptor inhibits proinflammatory cytokine-induced epithelial necroptosis in vitro: implications for preservation of intestinal barrier function. Am J Physiol Gastrointest Liver Physiol. (2025) 329:G261–9. doi: 10.1152/ajpgi.00086.2025, 40569574

[13] Attinkara R Mwinyi J Truninger K Regula J Gaj P Rogler G . Association of genetic variation in the NR1H4 gene, encoding the nuclear bile acid receptor FXR, with inflammatory bowel disease. BMC Res Notes. (2012) 5:461. doi: 10.1186/1756-0500-5-461, 22929053 PMC3517390

[14] Blake AB Guard BC Honneffer JB Lidbury JA Steiner JM Suchodolski JS. Altered microbiota, fecal lactate, and fecal bile acids in dogs with gastrointestinal disease. PLoS One. (2019) 14:e0224454. doi: 10.1371/journal.pone.0224454, 31671166 PMC6822739

[15] Wang S Martins R Sullivan MC Friedman ES Misic AM El-Fahmawi A . Diet-induced remission in chronic enteropathy is associated with altered microbial community structure and synthesis of secondary bile acids. Microbiome. (2019) 7:126. doi: 10.1186/s40168-019-0740-4, 31472697 PMC6717631

[16] Manchester AC Chow L Wheat W Dow S. Modulation of in vitro macrophage responses via primary and secondary bile acids in dogs. Animals. (2023) 13:3174. doi: 10.3390/ani13233714, 38067065 PMC10705343

[17] Sahoo DK Borcherding DC Chandra L Jergens AE Atherly T Bourgois-Mochel A . Differential transcriptomic profiles following stimulation with lipopolysaccharide in intestinal organoids from dogs with inflammatory bowel disease and intestinal mast cell tumor. Cancers (Basel). (2022) 14:3525. doi: 10.3390/cancers14143525, 35884586 PMC9322748

[18] Pratscher B Kuropka B Csukovich G Doulidis PG Spirk K Kramer N . Traces of canine inflammatory bowel disease reflected by intestinal organoids. Int J Mol Sci. (2024) 25:576. doi: 10.3390/ijms25010576, 38203746 PMC10778911

[19] Giaretta PR Rech RR Guard BC Blake AB Blick AK Steiner JM . Comparison of intestinal expression of the apical sodium-dependent bile acid transporter between dogs with and without chronic inflammatory enteropathy. J Vet Intern Med. (2018) 32:1918–26. doi: 10.1111/jvim.15332, 30315593 PMC6271328

[20] Fu T Li Y Oh TG Cayabyab F He N Tang Q . FXR mediates ILC-intrinsic responses to intestinal inflammation. Proc Natl Acad Sci USA. (2022) 119:e2213041119. doi: 10.1073/pnas.2213041119, 36508655 PMC9907109

[21] Stojancevic M Stankov K Mikov M. The impact of farnesoid X receptor activation on intestinal permeability in inflammatory bowel disease. Can J Gastroenterol. (2012) 26:631–7. doi: 10.1155/2012/538452, 22993736 PMC3441172

[22] Kliewer SA Mangelsdorf DJ. Bile acids as hormones: the FXR-FGF15/19 pathway. Dig Dis. (2015) 33:327–31. doi: 10.1159/000371670, 26045265 PMC4465534

[23] Nijmeijer RM Gadaleta RM van Mil SWC Bodegraven AA Crusius JBA Dijkstra G . Farnesoid X receptor (FXR) activation and FXR genetic variation in inflammatory bowel disease. PLoS One. (2011) 6:e23745. doi: 10.1371/journal.pone.0023745, 21887309 PMC3161760

[24] Negroni A Fiaschini N Palone F Vitali R Colantoni E Laudadio I . Intestinal inflammation alters the expression of hepatic bile acid receptors causing liver impairment. J Pediatr Gastroenterol Nutr. (2020) 71:189–96. doi: 10.1097/MPG.0000000000002759, 32404746

[25] Chiang JYL. Bile acid metabolism and signaling. Compr Physiol. (2013) 3:1191–212. doi: 10.1002/cphy.c120023, 23897684 PMC4422175

[26] Chiang JYL Pathak P Liu H Donepudi A Ferrell J Boehme S. Intestinal FXR and TGR5 signaling in metabolic regulation. Dig Dis. (2017) 35:241–5. doi: 10.1159/000450981, 28249273 PMC5470086

[27] Wilson A Wang Q Almousa AA Jansen LE Choi YH Schwarz UI . Genetic variation in the farnesoid X-receptor predicts Crohn’s disease severity in female patients. Sci Rep. (2020) 10:11725. doi: 10.1038/s41598-020-68686-9, 32678214 PMC7366697

[28] Klepsch V Moschen AR Tilg H Baier G Hermann-Kleiter N. Nuclear receptors regulate intestinal inflammation in the context of IBD. Front Immunol. (2019) 10:10. doi: 10.3389/fimmu.2019.01070, 31139192 PMC6527601

[29] Shea HC Head DD Setchell KDR Russell DW. Analysis of HSD3B7 knockout mice reveals that a 3alpha-hydroxyl stereochemistry is required for bile acid function. Proc Natl Acad Sci USA. (2007) 104:10–33. doi: 10.1073/pnas.0705089104, 17601774 PMC1913850

[30] Calzadilla N Comiskey SM Dudeja PK Saksena S Gill RK Alrefai WA. Bile acids as inflammatory mediators and modulators of intestinal permeability. Front Immunol. (2022) 13:13. doi: 10.3389/fimmu.2022.1021924, 36569849 PMC9768584

[31] Jahnel J Fickert P Hauer AC Högenauer C Avian A Trauner M. Inflammatory bowel disease alters intestinal bile acid transporter expression. Drug Metab Dispos. (2014) 42:1423–31. doi: 10.1124/dmd.114.058065, 24965812

[32] Manchester AC Ammons DT Lappin MR Dow S. Single cell transcriptomic analysis of the canine duodenum in chronic inflammatory enteropathy and health. Front Immunol. (2024) 15:15. doi: 10.3389/fimmu.2024.1397590, 38933260 PMC11199541

[33] Washabau RJ Day MJ Willard MD Hall EJ Jergens AE Mansell J . Endoscopic, biopsy, and histopathologic guidelines for the evaluation of gastrointestinal inflammation in companion animals. J Vet Intern Med. (2010) 24:10–26. doi: 10.1111/j.1939-1676.2009.0443.x, 20391635

[34] Prieto C Barrios D. RaNA-Seq: interactive RNA-Seq analysis from FASTQ files to functional analysis. Bioinformatics. (2019):btz854. doi: 10.1093/bioinformatics/btz854, 31730197

[35] Patro R Duggal G Love MI Irizarry RA Kingsford C. Salmon provides fast and bias-aware quantification of transcript expression. Nat Methods. (2017) 14:417–9. doi: 10.1038/nmeth.4197, 28263959 PMC5600148

[36] Galler AI Suchodolski JS Steiner JM Sung CH Hittmair KM Richter B . Microbial dysbiosis and fecal metabolomic perturbations in Yorkshire terriers with chronic enteropathy. Sci Rep. (2022) 12:12977. doi: 10.1038/s41598-022-17244-6, 35902689 PMC9334271

[37] Chiang JYL. Regulation of bile acid synthesis: pathways, nuclear receptors, and mechanisms. J Hepatol. (2004) 40:539–51. doi: 10.1016/j.jhep.2003.11.006, 15123373

