# Supplementary material for: Duodenal mucosal RNA-Seq identifies coordinated bile acid–axis transcriptional alterations in food-responsive enteropathy in dogs
Source: Front Vet Sci. 2026 Jun 11;13:1829399. doi: 10.3389/fvets.2026.1829399 (PMC13293934; doi:10.3389/fvets.2026.1829399)

Supplementary Figure S1A-C. Principal component analysis (PCA) of duodenal mucosal transcriptomes.

Samples are plotted along PC1 (65.76% variance explained) and PC2 (11.62% variance explained).

(A) Samples colored by group (FRE, orange; control, blue).
(B) Samples colored by age (years; blue-to-red gradient).
(C) Samples colored by body weight (kg; blue-to-red gradient).

PCA coordinates are identical across panels; only color coding differs.

Sample IDs are shown next to each point, and corresponding PCA coordinates and metadata are provided in Supplementary Table S3.

Supplementary Figure S1A

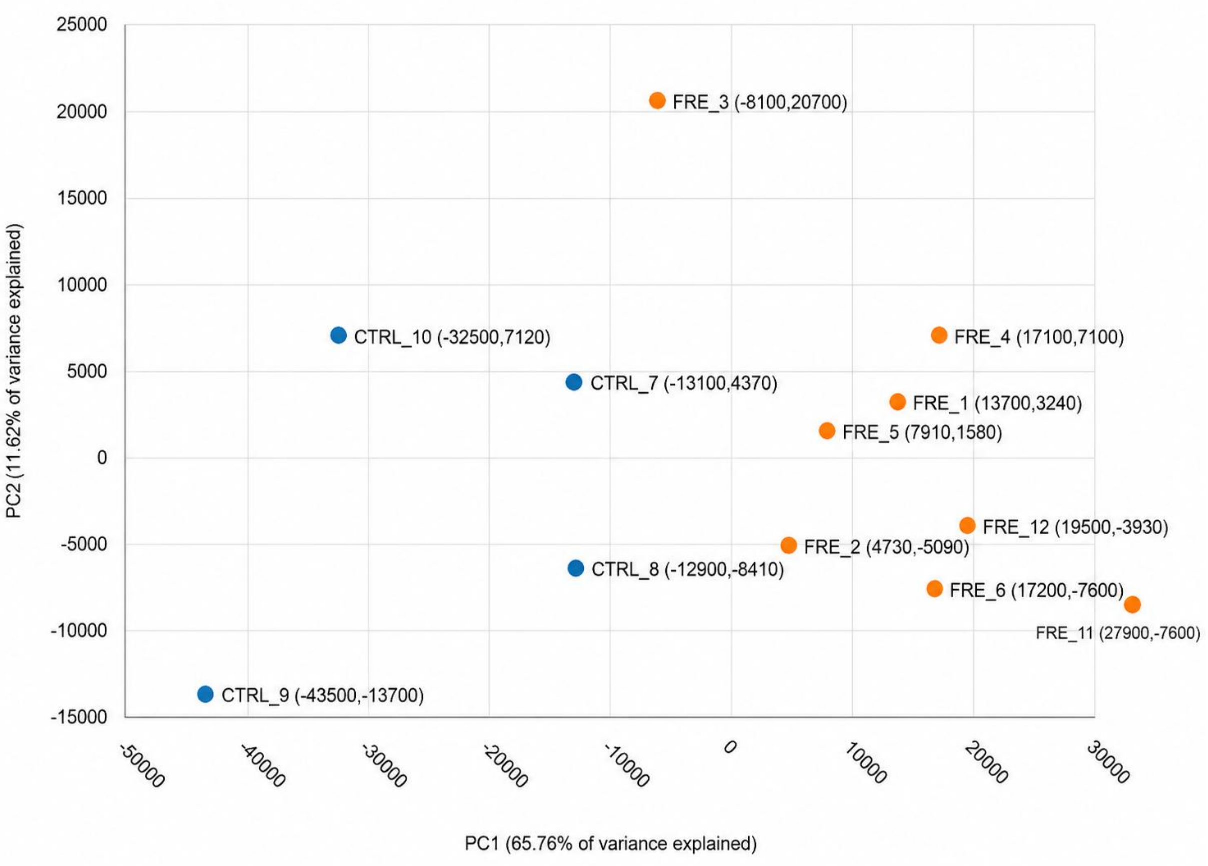

Supplementary Figure S1B

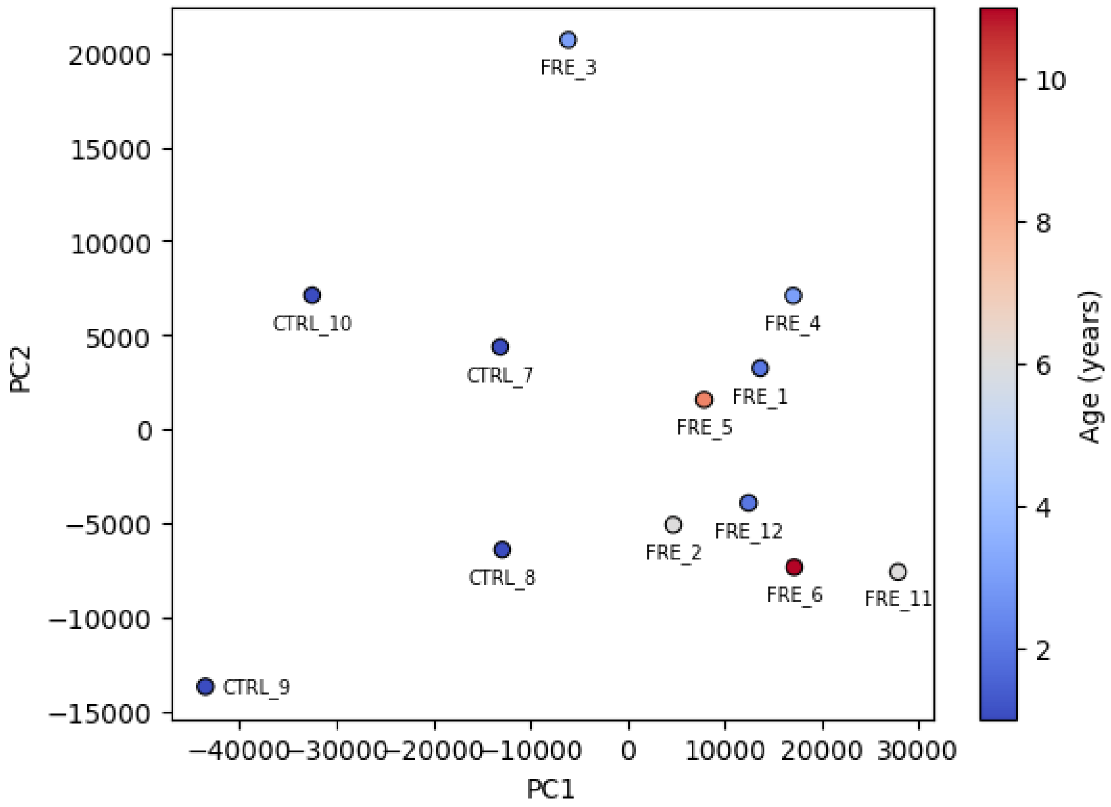

Supplementary Figure S1C

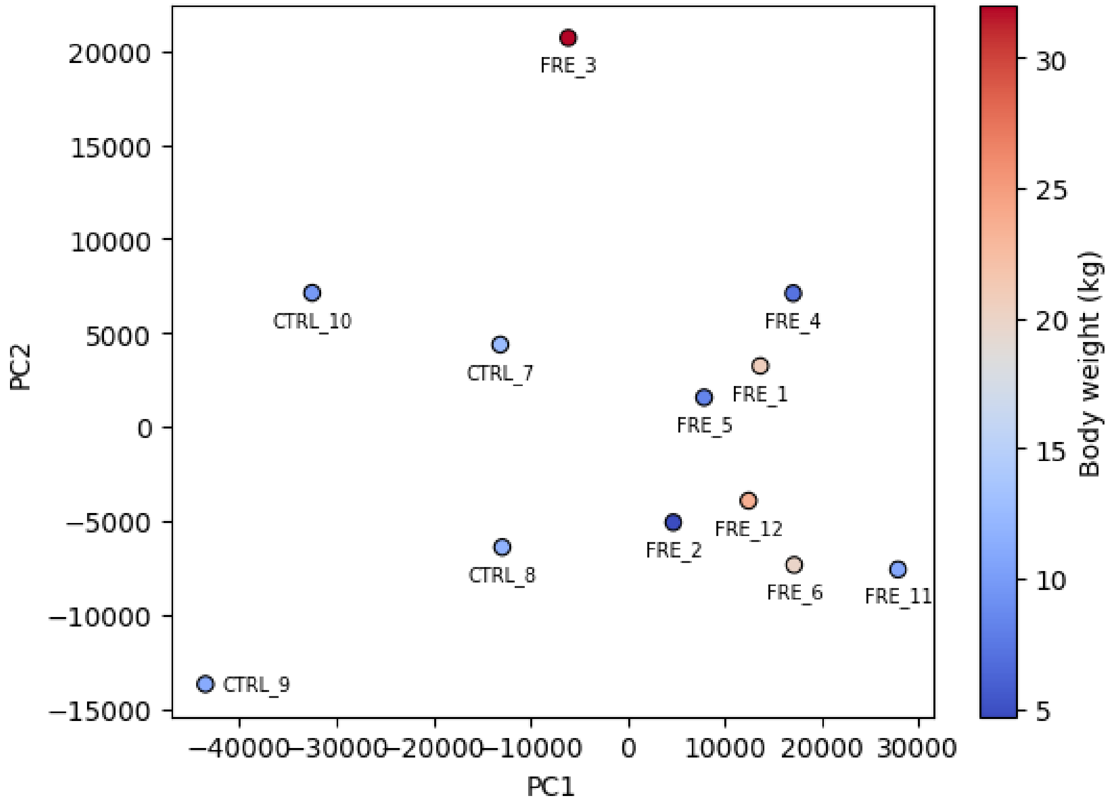

Supplement: Supplementary file 1 [file Image_1.pdf]
