# Supplementary material for: Duodenal mucosal RNA-Seq identifies coordinated bile acid–axis transcriptional alterations in food-responsive enteropathy in dogs
Source: Front Vet Sci. 2026 Jun 11;13:1829399. doi: 10.3389/fvets.2026.1829399 (PMC13293934; doi:10.3389/fvets.2026.1829399)

Supplementary Figure S2. Volcano plot of differential gene expression between FRE and control samples. Each point represents a gene plotted by log2 fold change (log2FC) and −log10 adjusted p-value (padj). Significantly differentially expressed genes (padj < 0.05) are shown in light pink, while non-significant genes are shown in grey. Bile acid–related genes are highlighted: significantly altered genes are shown in yellow, while non-significant genes (including NR1H4) are shown in grey. Gene labels indicate bile acid–related genes, with leader lines linking labels to their corresponding data points.

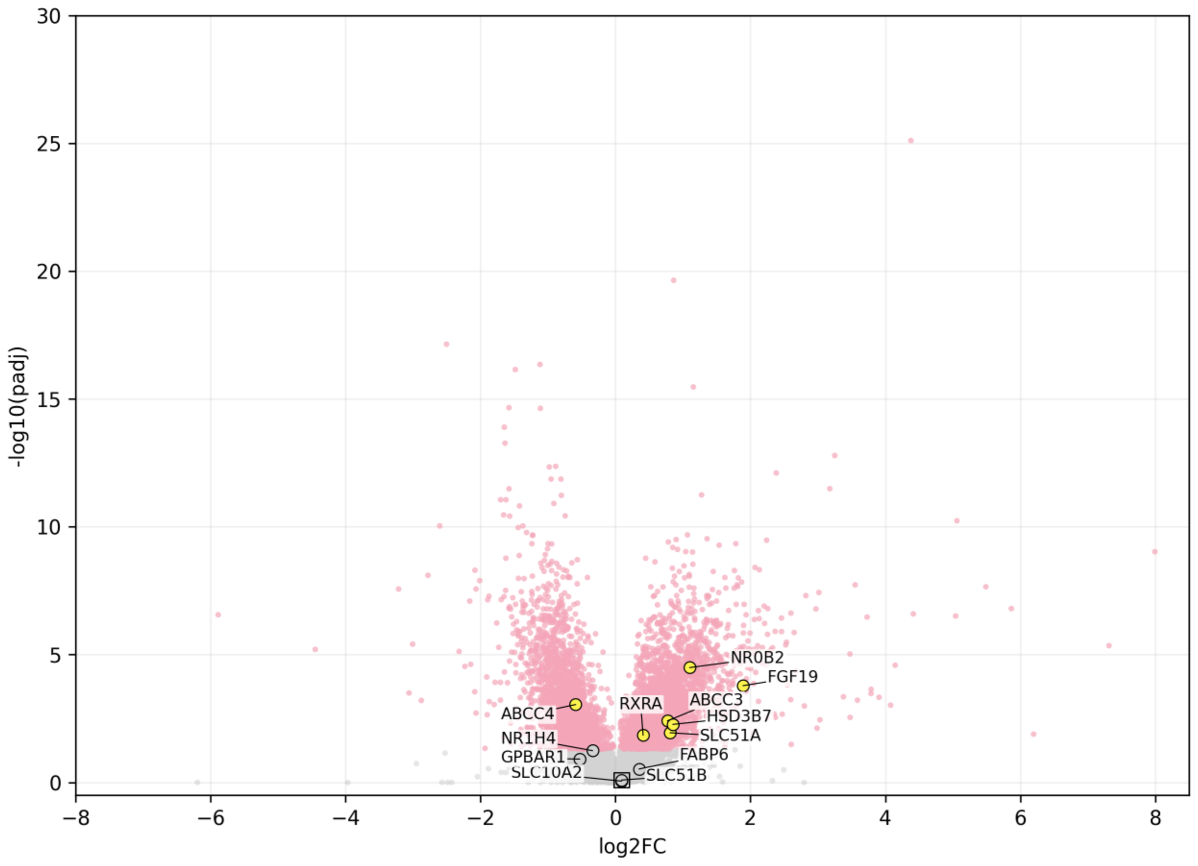

Supplement: Supplementary file 2 [file Image_2.pdf]
