# Supplementary material for: Duodenal mucosal RNA-Seq identifies coordinated bile acid–axis transcriptional alterations in food-responsive enteropathy in dogs
Source: Front Vet Sci. 2026 Jun 11;13:1829399. doi: 10.3389/fvets.2026.1829399 (PMC13293934; doi:10.3389/fvets.2026.1829399)

Supplementary Figure S3. KEGG Bile secretion pathway (cfa04976) highlighting differentially expressed bile acid–associated genes in dogs with food-responsive enteropathy (FRE). Pink indicates genes identified as differentially expressed; direction of change is described in the text.

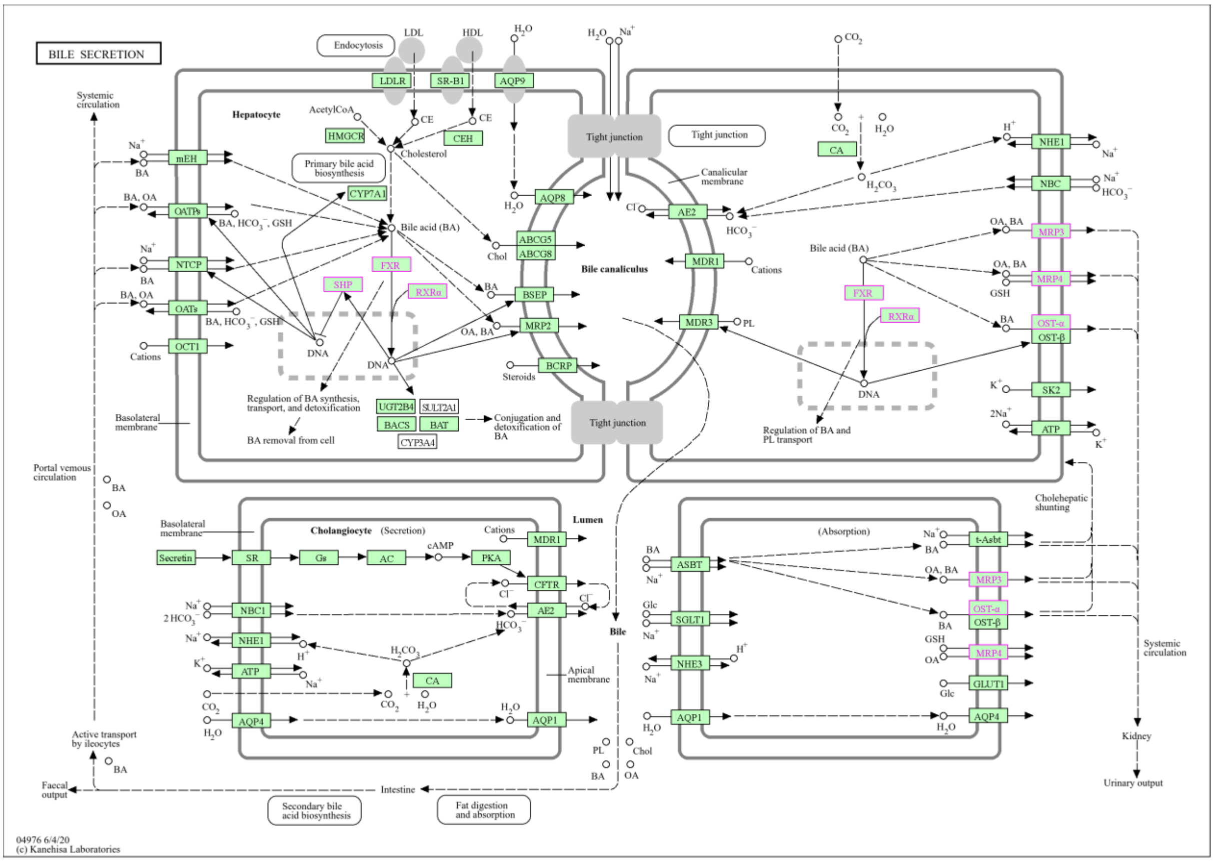

Supplement: Supplementary file 3 [file Image_3.pdf]
