# Supplementary material for: Duodenal mucosal RNA-Seq identifies coordinated bile acid–axis transcriptional alterations in food-responsive enteropathy in dogs
Source: Front Vet Sci. 2026 Jun 11;13:1829399. doi: 10.3389/fvets.2026.1829399 (PMC13293934; doi:10.3389/fvets.2026.1829399)
Supplement: Supplementary file 4 [file Image_4.pdf]

Supplementary Figure S4. KEGG Primary bile acid biosynthesis pathway (cfa00120) with annotation of HSD3B7, corresponding to EC 1.1.1.181. The pathway map illustrates the position of HSD3B7 with pink color within the canonical bile acid synthesis cascade; direction of change is described in the text.

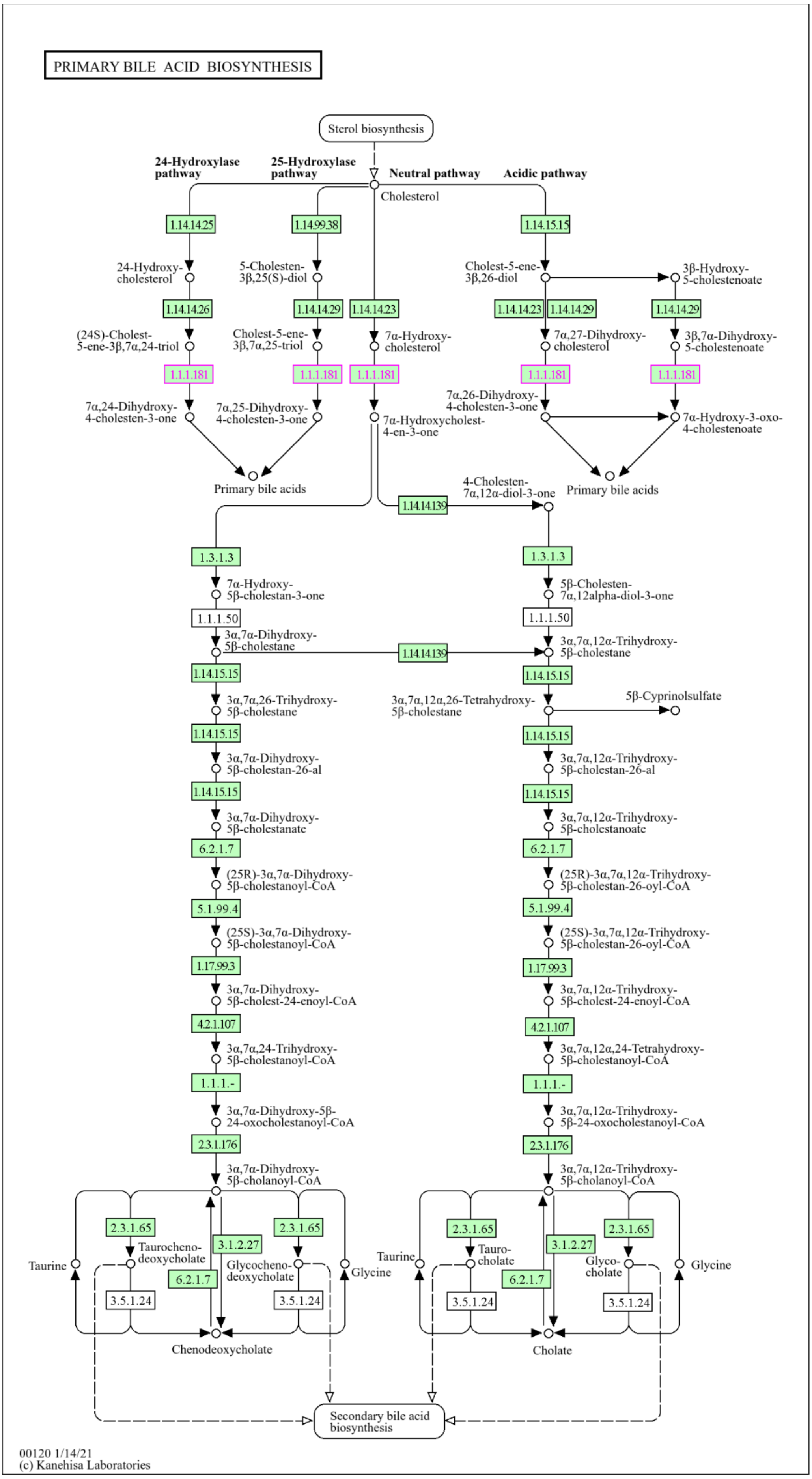
PRIMARY BILE ACID BIOSYNTHESIS
Sterol biosynthesis
24-Hydroxylase pathway
25-Hydroxylase pathway
Neutral pathway
Acidic pathway
Cholesterol
1.14.14.25
1.14.99.38
1.14.15.15
24-Hydroxy-cholesterol
5-Cholesten-3β,25(S)-diol
Cholest-5-ene-3β,26-diol
3β-Hydroxy-5-cholestenoate
1.14.14.26
1.14.14.29
1.14.14.23
1.14.14.23
1.14.14.29
1.14.14.29
(24S)-Cholest-5-ene-3β,7α,24-triol
Cholest-5-ene-3β,7α,25-triol
7α-Hydroxy-cholesterol
7α,27-Dihydroxy-cholesterol
3β,7α-Dihydroxy-5-cholestenoate
1.1.1.181
1.1.1.181
1.1.1.181
1.1.1.181
1.1.1.181
7α,24-Dihydroxy-4-cholesten-3-one
7α,25-Dihydroxy-4-cholesten-3-one
7α-Hydroxycholest-4-en-3-one
7α,26-Dihydroxy-4-cholesten-3-one
7α-Hydroxy-3-oxo-4-cholestenoate
Primary bile acids
Primary bile acids
4-Cholesten-7α,12α-diol-3-one
1.14.14.139
1.3.1.3
1.3.1.3
7α-Hydroxy-5β-cholestan-3-one
5β-Cholesten-7α,12alpha-diol-3-one
1.1.1.50
1.1.1.50
3α,7α-Dihydroxy-5β-cholestane
1.14.14.139
3α,7α,12α-Trihydroxy-5β-cholestane
1.14.15.15
1.14.15.15
3α,7α,26-Trihydroxy-5β-cholestane
3α,7α,12α,26-Tetrahydroxy-5β-cholestane
5β-Cyprinolsulfate
1.14.15.15
1.14.15.15
3α,7α-Dihydroxy-5β-cholestan-26-al
3α,7α,12α-Trihydroxy-5β-cholestan-26-al
1.14.15.15
1.14.15.15
3α,7α-Dihydroxy-5β-cholestanate
3α,7α,12α-Trihydroxy-5β-cholestanoate
6.2.1.7
6.2.1.7
(25R)-3α,7α-Dihydroxy-5β-cholestanoyl-CoA
(25R)-3α,7α,12α-Trihydroxy-5β-cholestan-26-oyl-CoA
5.1.99.4
5.1.99.4
(25S)-3α,7α-Dihydroxy-5β-cholestanoyl-CoA
(25S)-3α,7α,12α-Trihydroxy-5β-cholestan-26-oyl-CoA
1.17.99.3
1.17.99.3
3α,7α-Dihydroxy-5β-cholest-24-enoyl-CoA
3α,7α,12α-Trihydroxy-5β-cholest-24-enoyl-CoA
4.2.1.107
4.2.1.107
3α,7α,24-Trihydroxy-5β-cholestanoyl-CoA
3α,7α,12α,24-Tetrahydroxy-5β-cholestanoyl-CoA
1.1.1.-
1.1.1.-
3α,7α-Dihydroxy-5β-24-oxocholestanoyl-CoA
3α,7α,12α-Trihydroxy-5β-24-oxocholestanoyl-CoA
2.3.1.176
2.3.1.176
3α,7α-Dihydroxy-5β-cholanoyl-CoA
3α,7α,12α-Trihydroxy-5β-cholanoyl-CoA
2.3.1.65
2.3.1.65
3.1.2.27
Taurine
Taurocheno-deoxycholate
6.2.1.7
Glycocheno-deoxycholate
Glycine
3.5.1.24
3.5.1.24
Chenodeoxycholate
2.3.1.65
2.3.1.65
3.1.2.27
Taurine
Tauro-cholate
6.2.1.7
Glyco-cholate
Glycine
3.5.1.24
3.5.1.24
Cholate
Secondary bile acid biosynthesis
00120 1/14/21
(c) Kanehisa Laboratories
