# Supplementary material for: Duodenal mucosal RNA-Seq identifies coordinated bile acid–axis transcriptional alterations in food-responsive enteropathy in dogs
Source: Front Vet Sci. 2026 Jun 11;13:1829399. doi: 10.3389/fvets.2026.1829399 (PMC13293934; doi:10.3389/fvets.2026.1829399)
Supplement: Supplementary file 5 [file Table_1.docx]

**Supplementary Table 1.A.** Demographic characteristics of dogs included in the study. Breed, sex, age, and bodyweight of eight dogs with food-responsive enteropathy (FRE) and four healthy control beagles used for transcriptomic analysis. FRE dogs were client-owned animals, whereas control samples were obtained from purpose-bred research beagles without a history of gastrointestinal disease.

| Breed | Sex | Age (years) | Bodyweight (kg) | Group |
| --- | --- | --- | --- | --- |
| Miniature poodle | female spayed | 3 | 7.2 | FRE |
| Mixed breed | male castrated | 9 | 8.4 | FRE |
| Staffordshire bullterrier | female spayed | 11 | 19.9 | FRE |
| Australian shepherd | male intact | 2 | 20.6 | FRE |
| German shepherd | female spayed | 3 | 32 | FRE |
| Havanese dog | female spayed | 6 | 4.7 | FRE |
| Mixed breed | female intact | 2 | 23.5 | FRE |
| Mixed breed | male castrated | 6 | 10.9 | FRE |
| Beagle | male intact | 1 | 12.5 | Control |
| Beagle | male intact | 1 | 11.8 | Control |
| Beagle | female intact | 1 | 11.2 | Control |
| Beagle | female intact | 1 | 9.8 | Control |

**Supplementary Table 1.B.** Leading clinical signs and duration, CIBDAI score ([Canine Inflammatory Bowel Disease Activity Index](https://www.google.com/search?sca_esv=b1359834a2735cce&sxsrf=ANbL-n6ngaTM1EhFrfAQRaUcSdWZsUEwpA%3A1775927732561&q=Canine+Inflammatory+Bowel+Disease+Activity+Index&source=lnms&fbs=ADc_l-akmJ9clyHhwEynr9YRwEo_tYQUWp-_aNxOcHgKpLE-YUy1rF_kA3bn_mrSgXcgNhms40HIzKorfAboV7FAL0g4NuAevMWnqMN-rlw05BdG8zKPUc_-AzXQpP6wYaoI9WDsCP32C85iA-mOSR0nd0xC_cGGByJ1YS_Hb-fATDArJQWzE_UNRxJNfNei_E_fbi6vhtAvl68joZDJT9hKyfsXoIHmbw&sa=X&ved=2ahUKEwjG_bzrv-aTAxVBAhAIHbQqNjYQgK4QegQIARAC&biw=1600&bih=739&dpr=1&mstk=AUtExfBsPFPBSU4DyuAqhxzM2-BmmMTV9EUyvgedEr2KaWV-B_73UdfoPLViMFofi3Zkz1a6whclGYUV8trsO63QcBL6XhJ1VpbTiNibXT7ZnqOUbixk8ekH8YBNeTYYPX--FWbFkYeY6N7ERlt1RSItJQgpm6gLzXPSqjyEPFVXcbDzU2pMvHUQjlMAcUVTrqSn4FQ7QsBcoFQNvf_NQmg4BCvw1iMOEbLQsAqN5vW09tasrw&csui=3)), serum albumin, globulin and cholersterol levels of the FRE patients. SBD: small bowel diarrhoea. LBD: large bowel diarrhoea. Ref: reference range.

| Breed | Clinical signs and duration | CIBDAI | Albumin (g/dL) Ref: 2.3-4.1 | Globulin (g/dL) Ref: 2.5-4.5 | Cholesterol (mg/dL) Ref: 120-270 |
| --- | --- | --- | --- | --- | --- |
| Miniature poodle | vomiting, SBD; 5 months | 8 | 3.1 | 3.9 | 198 |
| Mixed breed | vomiting, SBD, LBD, weight loss; 8 months | 5 | 2.6 | 3.2 | 178 |
| Staffordshire bullterrier | vomiting, SBD, LBD; 4 months | 5 | 2.8 | 4.8 | 226 |
| Australian shepherd | vomiting, SBD, LBD; 6 months | 7 | 3.2 | 4.6 | 230 |
| German shepherd | SBD, weight loss; 1 year | 6 | 2.4 | 3.1 | 150 |
| Havanese dog | vomiting, SBD; 1.5 years | 8 | 2.7 | 3.7 | 185 |
| Mixed breed | SBD, weight loss, hyporexia; 4 months | 4 | 2.9 | 3.0 | 217 |
| Mixed breed | vomiting, SBD, weight loss; 4 months | 6 | 3.0 | 4.0 | 243 |
