# Supplementary material for: Duodenal mucosal RNA-Seq identifies coordinated bile acid–axis transcriptional alterations in food-responsive enteropathy in dogs
Source: Front Vet Sci. 2026 Jun 11;13:1829399. doi: 10.3389/fvets.2026.1829399 (PMC13293934; doi:10.3389/fvets.2026.1829399)

**Supplementary Table S2.** Sample-level sequencing quality metrics for all analyzed samples. For each sample, total fragment counts (library size), the number and proportion of matched fragments (mapping rate), proportion of properly paired fragments, and GC content (%) are reported. Sequencing quality metrics were consistent across samples, with mapping rates ranging from 68.8% to 71.1% and properly paired fragments exceeding 90% in all cases.


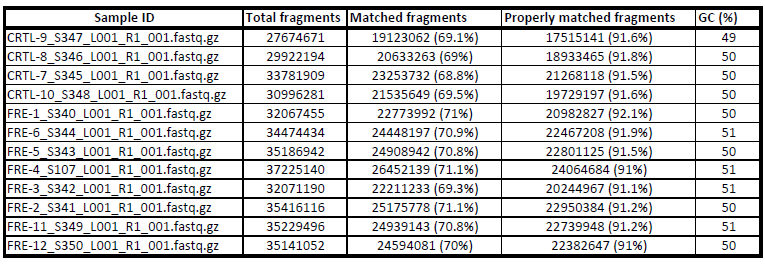

Supplement: Supplementary file 6 [file Table_2.docx]
