# Supplementary material for: Duodenal mucosal RNA-Seq identifies coordinated bile acid–axis transcriptional alterations in food-responsive enteropathy in dogs
Source: Front Vet Sci. 2026 Jun 11;13:1829399. doi: 10.3389/fvets.2026.1829399 (PMC13293934; doi:10.3389/fvets.2026.1829399)
Supplement: Supplementary file 7 [file Table_3.docx]

**Supplementary Table S3.** Sample ID, breed, sex, age, body weight, group assignment, and PCA1 and PCA2 coordinates of eight dogs with food-responsive enteropathy (FRE) and four healthy control (CTRL) beagles used for transcriptomic analysis are provided. FRE dogs were client-owned animals, whereas control samples were obtained from purpose-bred research beagles without a history of gastrointestinal disease. The inclusion of PCA coordinates allows direct linkage between individual samples, metadata, and their distribution in PCA space for independent evaluation of potential biological and technical influences on global transcriptional patterns.

| **ID** | **Breed** | **Sex** | **Age (years)** | **Bodyweight (kg)** | **Group** | **PC1** | **PC2** |
| --- | --- | --- | --- | --- | --- | --- | --- |
| 4 | Miniature poodle | female spayed | 3 | 7,2 | FRE | 17100 | 7100 |
| 5 | Mixed breed | male castrated | 9 | 8,4 | FRE | 7910 | 1560 |
| 6 | Staffordshire bullterrier | female spayed | 11 | 19,9 | FRE | 17200 | -7350 |
| 1 | Australian shepherd | male intact | 2 | 20,6 | FRE | 13700 | 3240 |
| 3 | German shepherd | female spayed | 3 | 32 | FRE | -6100 | 20700 |
| 2 | Havanese dog | female spayed | 6 | 4,7 | FRE | 4730 | -5090 |
| 12 | Mixed breed | female intact | 2 | 23,5 | FRE | 12500 | -3930 |
| 11 | Mixed breed | male castrated | 6 | 10,9 | FRE | 27900 | -7600 |
| 7 | Beagle | male intact | 1 | 12,5 | CTRL | -13100 | 4370 |
| 8 | Beagle | male intact | 1 | 11,8 | CTRL | -12900 | -6410 |
| 9 | Beagle | female intact | 1 | 11,2 | CTRL | -43500 | -13700 |
| 10 | Beagle | female intact | 1 | 9,8 | CTRL | -32500 | 7120 |
