# Supplementary material for: Duodenal mucosal RNA-Seq identifies coordinated bile acid–axis transcriptional alterations in food-responsive enteropathy in dogs
Source: Front Vet Sci. 2026 Jun 11;13:1829399. doi: 10.3389/fvets.2026.1829399 (PMC13293934; doi:10.3389/fvets.2026.1829399)
Supplement: Supplementary file 8 [file Table_4.docx]

**Supplementary Table S4.** Results of gene set enrichment analysis (GSEA) based on KEGG pathways in duodenal mucosal samples from dogs with food-responsive enteropathy (FRE) and healthy controls. Pathways are ranked according to normalized enrichment score (NES). Statistical significance was assessed using nominal p-values and adjusted p-values (padj; Benjamini–Hochberg correction). Positive NES values indicate enrichment in FRE samples relative to controls.

| **Pathway_ID** | **Pathway** | **Database** | **NES** | **p-value** | **padj** |
| --- | --- | --- | --- | --- | --- |
| cfa04975 | Fat digestion and absorption | KEGG | 2,345 | 1.7e-4 | 0,005 |
| cfa00020 | Citrate cycle (TCA cycle) | KEGG | 2,182 | 1.8e-4 | 0,005 |
| cfa04140 | Autophagy - animal | KEGG | 2,142 | 1.6e-4 | 0,005 |
| cfa04152 | AMPK signaling pathway | KEGG | 2,107 | 1.6e-4 | 0,005 |
| cfa01200 | Carbon metabolism | KEGG | 2,019 | 1.6e-4 | 0,005 |
| cfa04137 | Mitophagy - animal | KEGG | 1,99 | 1.7e-4 | 0,005 |
| cfa04146 | Peroxisome | KEGG | 1,978 | 1.6e-4 | 0,005 |
| cfa04530 | Tight junction | KEGG | 1,936 | 1.5e-4 | 0,005 |
| **cfa04976** | **Bile secretion** | **KEGG** | **1,935** | **1.7e-4** | **0,005** |
| cfa04012 | ErbB signaling pathway | KEGG | 1,927 | 1.7e-4 | 0,005 |
| cfa04141 | Protein processing in endoplasmic reticulum | KEGG | 1,914 | 1.5e-4 | 0,005 |
| cfa03320 | PPAR signaling pathway | KEGG | 1,873 | 3.3e-4 | 0,006 |
| cfa04070 | Phosphatidylinositol signaling system | KEGG | 1,865 | 1.6e-4 | 0,005 |
| cfa04972 | Pancreatic secretion | KEGG | 1,842 | 1.6e-4 | 0,005 |
| cfa04144 | Endocytosis | KEGG | 1,69 | 1.5e-4 | 0,005 |
| cfa04068 | FoxO signaling pathway | KEGG | 1,649 | 3.2e-4 | 0,006 |
| cfa04659 | Th17 cell differentiation | KEGG | -1,725 | 2.6e-4 | 0,005 |
| cfa04658 | Th1 and Th2 cell differentiation | KEGG | -1,757 | 2.5e-4 | 0,005 |
| cfa04064 | NF-kappa B signaling pathway | KEGG | -1,886 | 2.6e-4 | 0,005 |
| cfa04610 | Complement and coagulation cascades | KEGG | -2,06 | 2.5e-4 | 0,005 |
| cfa04514 | Cell adhesion molecules (CAMs) | KEGG | -2,114 | 2.7e-4 | 0,005 |
| cfa05321 | Inflammatory bowel disease (IBD) | KEGG | -2,201 | 2.4e-4 | 0,005 |
| cfa04672 | Intestinal immune network for IgA production | KEGG | -2,318 | 2.4e-4 | 0,005 |
| cfa04060 | Cytokine-cytokine receptor interaction | KEGG | -2,372 | 3.0e-4 | 0,005 |
| cfa_M00177 | Ribosome, eukaryotes | KEGG | -2,556 | 2.5e-4 | 0,005 |
