# Supplementary material for: Duodenal mucosal RNA-Seq identifies coordinated bile acid–axis transcriptional alterations in food-responsive enteropathy in dogs
Source: Front Vet Sci. 2026 Jun 11;13:1829399. doi: 10.3389/fvets.2026.1829399 (PMC13293934; doi:10.3389/fvets.2026.1829399)

**Supplementary Table S5. Sensitivity (subsampling) analyses of FRE samples.** Subsampling analyses were performed to assess the impact of age and body size differences between groups. A “young FRE” subset (dogs aged 2–3 years) and a more restrictive subset excluding a high body-weight individual were analyzed. Across subsets, PCA structure and enrichment statistics varied due to reduced sample size; however, group separation was retained and bile acid–related pathway signals remained detectable by ORA or GSEA. These analyses are presented as exploratory sensitivity checks and are not intended to adjust for confounding.


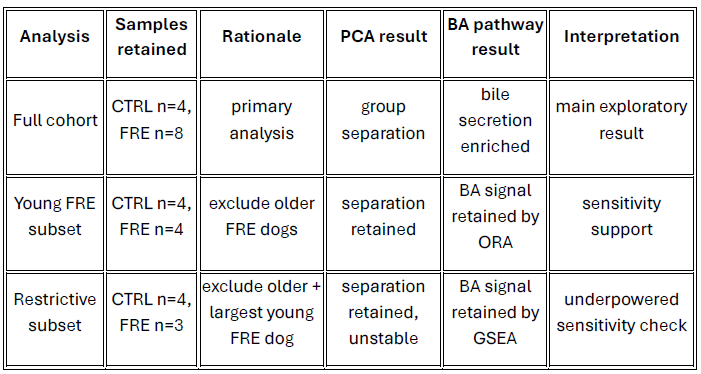

Supplement: Supplementary file 9 [file Table_5.docx]
