# Supplementary material for: Duodenal mucosal RNA-Seq identifies coordinated bile acid–axis transcriptional alterations in food-responsive enteropathy in dogs
Source: Front Vet Sci. 2026 Jun 11;13:1829399. doi: 10.3389/fvets.2026.1829399 (PMC13293934; doi:10.3389/fvets.2026.1829399)
Supplement: Supplementary file 10 [file Table_6.docx]

**Supplementary Table S6.** Group-wise mean raw read counts and transcripts per million (TPM) values for selected bile acid–associated genes in duodenal mucosal samples from healthy controls (CTRL) and dogs with food-responsive enteropathy (FRE).

| **Ensembl ID** | **Gene symbol** | **Mean raw counts (CTRL)** | **Mean raw counts (FRE)** | **Mean TPM (CTRL)** | **Mean TPM (FRE)** |
| --- | --- | --- | --- | --- | --- |
| ENSCAFG00000012261.3 | NR0B2 | 78.0 | 250.6 | 1.306 | 3.935 |
| ENSCAFG00000010693.2 | FGF19 | 0.5 | 25.4 | 0.065 | 2.937 |
| ENSCAFG00000017201.4 | ABCC3 | 2148.5 | 3481.4 | 11.277 | 19.935 |
| ENSCAFG00000016770.3 | HSD3B7 | 948.5 | 1708.8 | 27.635 | 50.711 |
| ENSCAFG00000005433.3 | ABCC4 | 867.5 | 556.6 | 6.722 | 4.128 |
| ENSCAFG00000012955.3 | SLC51A | 647.0 | 1211.0 | 23.420 | 41.855 |
| ENSCAFG00000019838.3 | RXRA | 3371.5 | 4765.1 | 28.478 | 38.203 |
| ENSCAFG00000006835.4 | NR1H4 | 573.4 | 452.1 | 14.325 | 11.125 |
| ENSCAFG00000014623.3 | GPBAR1 | 20.1 | 9.1 | 1.146 | 0.482 |
| ENSCAFG00000017275.3 | FABP6 | 0.0 | 1.1 | 0.000 | 0.275 |
| ENSCAFG00000017121.3 | SLC51B | 543.2 | 594.8 | 135.040 | 144.101 |
| ENSCAFG00000006008.3 | SLC10A2 | 11.0 | 13.6 | 0.457 | 0.550 |
